# OVA-induced airway hyperresponsiveness alters murine heart rate variability and body temperature

**DOI:** 10.3389/fphys.2012.00456

**Published:** 2012-12-04

**Authors:** N. J. Domnik, G. Seaborn, S. G. Vincent, S. G. Akl, D. P. Redfearn, J. T. Fisher

**Affiliations:** ^1^Department of Biomedical and Molecular Sciences (Physiology Program), Queen's UniversityKingston, ON, Canada; ^2^School of Computing, Queen's UniversityKingston, ON, Canada; ^3^Medicine (Divisions of Cardiology), Queen's UniversityKingston, ON, Canada; ^4^Medicine (Divisions of Respirology), Queen's UniversityKingston, ON, Canada

**Keywords:** airway hyperresponsiveness, mouse, ovalbumin, heart rate variability, autonomic control, allergic sensitization, asthma, cardiovascular comorbidity

## Abstract

Altered autonomic (ANS) tone in chronic respiratory disease is implicated as a factor in cardiovascular co-morbidities, yet no studies address its impact on cardiovascular function in the presence of murine allergic airway (AW) hyperresponsiveness (AHR). Since antigen (Ag)-induced AHR is used to model allergic asthma (in which ANS alterations have been reported), we performed a pilot study to assess measurement feasibility of, as well as the impact of allergic sensitization to ovalbumin (OVA) on, heart rate variability (HRV) in a murine model. Heart rate (HR), body temperature (T_B_), and time- and frequency-domain HRV analyses, a reflection of ANS control, were obtained in chronically instrumented mice (telemetry) before, during and for 22 h after OVA or saline aerosolization in sensitized (OVA) or Alum adjuvant control exposed animals. OVA mice diverged significantly from Alum mice with respect to change in HR during aerosol challenge (*P* < 0.001, Two-Way ANOVA; HR max change Ctrl = +80 ± 10 bpm vs. OVA = +1 ± 23 bpm, mean ± SEM), and displayed elevated HR during the subsequent dark cycle (*P* = 0.006). Sensitization decreased the T_B_ during aerosol challenge (*P* < 0.001). Sensitized mice had decreased HRV *prior* to challenge (SDNN: *P* = 0.038; Low frequency (LF) power: *P* = 0.021; Low/high Frequency (HF) power: *P* = 0.042), and increased HRV during Ag challenge (RMSSD: *P* = 0.047; pNN6: *P* = 0.039). Sensitized mice displayed decreased HRV subsequent to OVA challenge, primarily in the dark cycle (RMSSD: *P* = 0.018; pNN6: *P* ≤ 0.001; LF: *P* ≤ 0.001; HF: *P* = 0.040; LF/HF: *P* ≤ 0.001). We conclude that implanted telemetry technology is an effective method to assess the ANS impact of allergic sensitization. Preliminary results show mild sensitization is associated with reduced HRV and a suppression of the acute T_B_-response to OVA challenge. This approach to assess altered ANS control in the acute OVA model may also be beneficial in chronic AHR models.

## Introduction

Asthma is an inflammatory condition of the airways (AW) characterized by airway hyperresponsiveness (AHR), inflammatory cell infiltration and reversible bronchoconstriction, which, on a chronic basis, progresses to AW remodeling (Boulet et al., 1999; Holgate, 2008; Lougheed et al., 2010). Asthma is often modeled in animals through allergic induction of AHR, which is characterized by a decrease in the bronchoconstrictive threshold and an increase in the maximal response generated for bronchoconstrictive stimuli (Boulet et al., 1999; Kumar et al., 2008; Allen et al., 2009). Patients with asthma show altered heart rate variability (HRV) profiles (Tokuyama et al., 1985; Lewis et al., 2006). In general, decreased HRV is associated with decreased cardiovascular prognosis (Chattipakorn et al., 2007). Although HRV analysis has been successfully adopted for use in murine models, to our knowledge this tool has not been applied to models of AHR (Thireau et al., 2008). Further, the mechanism(s) responsible for alterations in HRV and the time-course of their development in asthma are unknown. We addressed the hypothesis that changes in autonomic control, particularly HRV, are initiated early in the development of AHR. We assessed the utility of a chronically instrumented murine model of acute ovalbumin (OVA) sensitization, in which we investigated the effect of sensitization and antigen (Ag; i.e., OVA) challenge on the regulation of heart rate (HR) using time- and frequency-domain HRV analysis of ECG, as well as on core body temperature (T_B_) and activity between groups.

## Materials and methods

All experiments were approved by the Queen's Animal Care Committee and performed according to the guidelines of the Canadian Council of Animal Care.

### Protocol

We employed an OVA sensitization model (Walker et al., 2003; Kumar et al., 2008) in which male C57BL/6 mice (5 weeks old upon arrival; Charles River Canada) were randomly assigned to either OVA or aluminum hydroxide adjuvant control (Alum) groups. Mice were surgically implanted with sterile i.p., radiotelemetry devices (TA 10 ETA-F20; DSI International, St. Paul, MN) and allowed a 2-weeks recovery period to reestablish circadian variation [unpublished observations; (Thireau et al., 2008)]. Body weights *prior* to OVA inhalational challenge (mean ± SD) were not different between groups; 35.4 ± 2.0 g and 33.4 ± 3.8 g for OVA and Alum groups, respectively (*P* > 0.05). Animals were housed in a 12-h light/dark cycle (08:00–20:00 h light, 20:00–08:00 h dark) environment with an ambient temperature of 22°C, and *ad libitum* access to food and water.

**Figure 1:**
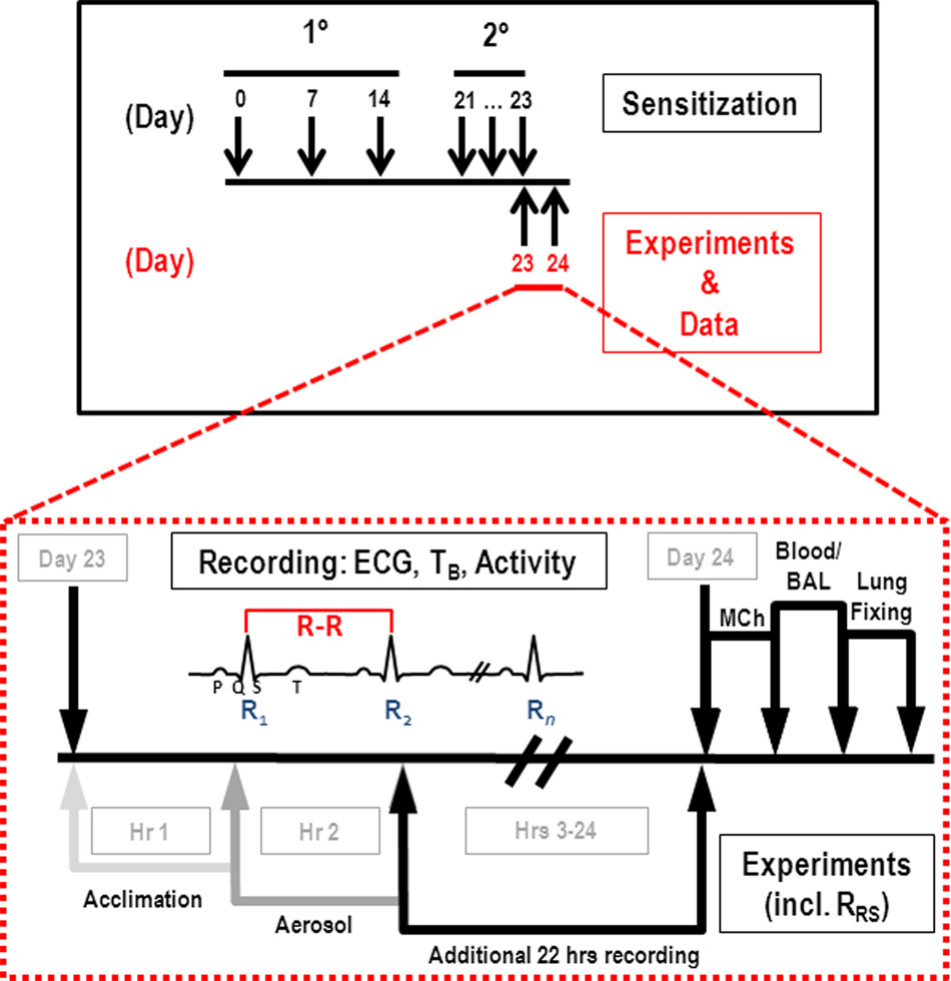
**Sensitization and experimental protocols.** Primary (i.p., injection) and secondary (aerosol) sensitizations occurred as indicated in the upper panel; expanded view comprises the third aerosol challenge (OVA or saline) on day 23. HR, T_B_, and activity data were collected on day 23 as indicated for 1 h *prior* to aerosol (light gray arrow), 1 h during aerosol (gray arrow), and 22 h post-challenge (black arrow). Subsequently, MCh challenge (R_RS_) was performed, and blood and BAL samples collected for analysis of inflammatory cells.

Sensitization, OVA challenge and data collections were staggered among the mice to accommodate *in vivo* physiologic measurements of AHR. The protocol consisted of 3 weeks of primary sensitization (or Alum control) followed by secondary sensitization and exposure to aerosolized OVA solution or sterile saline (Figure 1).

### Primary sensitization

Primary Sensitization consisted of i.p., injections (0.2 ml) of OVA suspended in aluminum hydroxide adjuvant (OVA group: 50 μl of 1 mg/ml A5503 Albumin Grade V + 80 μl of 13 mg/ml A8222 Aluminum Hydroxide Gel “Alhydrogel,” both Sigma-Alrich Inc., St. Louis, MO, + 870 μl sterile saline) or aluminum hydroxide adjuvant in saline (Alum group: 77 μl of 13 mg/ml A8222 Aluminum Hydroxide Gel “Alhydrogel,” Sigma-Alrich Inc., St. Louis, MO, + 923 μl sterile saline) administered once weekly for 3 weeks. Injections were given at consistent times of day and by the same individual to minimize intra-procedural variability.

### Secondary aerosol challenge

Mice were exposed to aerosolized challenges (60 min) on 3 consecutive days at approximately the same time each day (within 40 min). Solutions (1% weight/volume A5503 Albumin Grade V OVA in sterile saline and sterile saline for OVA and Alum groups, respectively,) were aerosolized by an ultrasonic nebulizer (Ultrasonic 2000 by Nouvag Dental and Medical Equipment; Goldach, Switzerland; particle size = 3.8–4.0 μm, chamber airflow = 700 ml/min). Aerosolized Ag challenges were performed in sealed 1.6 L Plexiglas chambers.

On the third aerosol exposure day (day 23 of protocol), telemetry data for ECG waveform, T_B_ and activity data were acquired during acclimation to the exposure-chamber (60 min *prior* to OVA/saline challenge), aerosolization (60 min of challenge) and for a subsequent 22 h post- challenge. Activity represents the relative locomotive activity of each animal, measured on the basis of the strength of the transmitted signal (telemetry device) to the Data Exchange Matrix; distance and speed of movement (“movement” includes orientation and distance) are considered in the calculation of activity units.

Data were sampled for 3 min on a 10 min interval schedule (ECG at 2k samples/s; T_B_ at 250 samples/s and activity at 64 samples/s; Dataquest ART Software, DSI International; St. Paul, MN). Temporally-synchronized video was monitored to provide visual surveillance of each mouse during the hour of aerosol challenge (15 frames/s).

### *In vivo* airway challenge

Following the telemetry protocol mice were anesthetized (sodium pentobarbital i.p., 60 mg/kg; dilution = 30 mg/ml), a tracheal cannula inserted and mechanical ventilation initiated (peak tracheal pressure: circa 7.5 cm H_2_O, respiratory frequency: 120 breaths/min; MOD.RV5 Ventilator, Voltek Enterprises Inc., Toronto, ON). This level of ventilation abolished respiratory efforts *prior* to administration of pancuronium bromide. The right jugular vein was catheterized to allow for intra-venous administration of supplemental anesthetic, pancuronium bromide (0.25 mg/kg) and methacholine (MCh). Arterial blood pressure was measured via a 1F Mikro-Tip Catheter Pressure Transducer (Millar: Sensors, Systems, Solutions; Houston, TX) inserted into the aortic arch via the left carotid artery.

Logarithmic dose-response curves for MCh were generated for all mice (dosage = 3 μg/kg, 10 μg/kg, 30 μg/kg, 100 μg/kg). Doses were administered at 15 min intervals, and were preceded by hyperinflation (two breaths) and a 2-min post-inflation period to control volume history. Following 30 s of baseline recording, MCh was injected via the jugular catheter, and responses over 100 s were recorded (ECG, blood pressure, transrespiratory system pressure, tidal volume [V_T_], and flow). Total respiratory resistance (R_RS_) was calculated on a breath-by-breath basis according to the method of Ewart et al. (1995) as measured using a ventilator technique described by Volgyesi et al. (2000). All signals were acquired digitally (SPIKE2 software, Cambridge Electronic Design, Cambridge, UK), and R_RS_ was defined as the ratio of the resistive AW pressure (peak P_AW–_plateau P_AW_; cm H_2_O) to flow, defined by V_T_ over inspiratory time (V_T_/T_I_; ml/s). Maximum R_RS_ values for each dose of MCh were reported.

### Inflammatory cell analysis

Bronchioalveolar lavage (BAL) samples for total white blood cell (WBC) count were centrifuged (5 min at 12,000 RPM), the supernatant removed and red blood cell lysis achieved through resuspension of the pellet in 1 ml lysis solution buffer (Tris/Amm. Cl). The solution was re-spun and the supernatant discarded. Following resuspension of the pellet in 1 ml sterile, buffered saline, total cell count was taken using a hemocytometer (10 μl fluid loaded). Data were reported as cells/ml.

BAL samples for differential WBC analysis (100 μl resuspended BAL; above) were loaded onto assembled cytospin cassettes along with 50 μl debris-free fetal calf serum. Samples were centrifuged (3 min at 800 RPM; Shandon Cytospin, Thermo Scientific, Kalamazoo, MI) and cells were allowed to dry ≥ 15 min before staining using the Protocol Hema3 Staining Kit (Fisher Scientific Co., Kalamazoo, MI). Differential counts (results reported in percent of total WBC population; minimum 200 cells total) were obtained for macrophages, neutrophils, lymphocytes, eosinophils, and AW epithelial cells.

Blood samples for total WBC count (20 μl) were mixed with 190 μl lysis solution buffer (Tris/Amm.Cl) and allowed to react for 10 min. Cells were counted through use of a hemocytometer (10 μl loaded) and results reported as cells/μl. Standard smears of the differential WBC count samples were made and allowed to dry on glass slides using 10 μl of well-mixed blood. Slides were stained using the Protocol HEMA3 kit and 100 circulating WBCs were differentiated, with results being reported as percentage of total WBCs.

### Statistical analysis

Statistical analyses were performed using SigmaStat 3.0 (Systat Software Inc., San Jose, CA). Parametric statistical analyses consisted of paired *t*-tests or One-Way and Two-Way analysis of variance (ANOVA) as indicated. If the assumptions for parametric tests were not met, ranked data were used in non-parametric analyses. *Post-hoc* tests (Holm-Sidak) were performed as appropriate. A significant difference was defined as *P* < 0.05.

### HRV analysis

Time- and frequency-domain HRV analyses were based on the standards developed by Thireau et al. for murine models (Thireau et al., 2008). Raw ECG data were analyzed manually for peak detection of R waves in order to obtain R-R, or inter-beat, intervals (IBI), which were exported into Excel for time domain analysis. Frequency domain analyses were performed offline using Matlab (MATLAB/R2010a; The MathWorks Inc., Natick, MA). Manual selection ensured that only true sinus beats were included in analysis and circumvented analytical issues associated with automated software (Torbey et al., 2012). This procedure removed the need to exclude beats outside two standard deviations of the mean, a filtering method generally employed to exclude non-sinus events (Thireau et al., 2008).

Time domain analysis was performed for all 3-min segments by calculating standard deviation of all normal R-R intervals (SDNN), square root of the mean of the sum of squares of successive differences between normal R-R intervals (RMSSD), and percentage of consecutive R-R intervals differing by more than 6 ms(pNN6) (Thireau et al., 2008). Comparisons were made between groups for all 3-min segments during acclimatization and aerosolization, and on an hourly basis for post-aerosolization data spanning 3–24 h of the protocol (each hour consisted of the mean of the six data points; i.e., one 3-min segment per 10 min).

In order to gain insight into the respective contributions of the sympathetic and parasympathetic nervous systems to heart rhythm, frequency domain HRV analysis was executed by fast Fourier transformation (FFT). R-R interval series were re-sampled to a 20 Hz inter-beat time series, mean-detrended, and Hamming windowed. The squared magnitudes of the discrete Fourier transform of the segments were averaged to form power spectral density. The frequency domain HRV parameters computed for all 3-min segments were: low frequency power (LF; 0.15–1.5 Hz), reflecting sympathetic activity; high frequency power (HF; 1.5–5 Hz), reflecting parasympathetic activity; and the LF/HF power ratio (Thireau et al., 2008). Although an LF band of 0.4–1.5 Hz has been recommended by numerous researchers (Wickman et al., 1998; Gehrmann et al., 2000; Pelat et al., 2003; Williams et al., 2003; Duan et al., 2007), we selected 0.15–1.5 Hz, as recommended for murine models by Thireau et al. (2008).

We performed further experiments on an additional group of mice given identical sensitization and exposure protocols to those outlined above. In these mice, the bronchoconstrictive effect of bolus right heart injection of 50 mg/kg OVA, as well as the bronchoconstrictive response to increasing doses of inhaled MCh, was measured.

## Results

**Figure 2:**
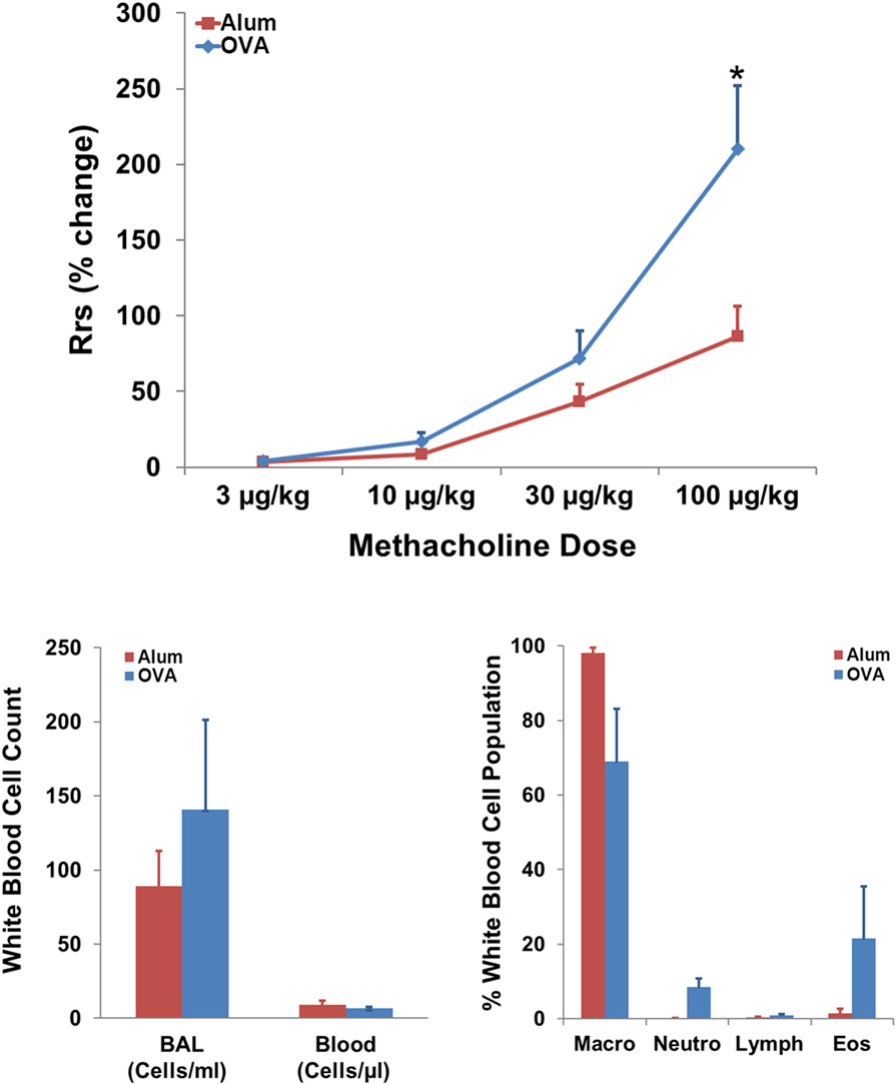
**Effect of sensitization on AHR and pulmonary WBC profile. Top panel:** OVA sensitization resulted in AHR, measured as an increased percent change in R_RS_ in response to MCh (*t*-test; *P* < 0.05). **Bottom panels:** Effect of OVA sensitization on systemic blood and BAL white blood cell count and BAL differential WBC profile. Non-significant trends toward increased localized pulmonary inflammation, as reflected by modest eosinophilia (Eos) in the OVA group, were observed in analyses of net WBC (bottom left) and white blood cell population breakdown (right panel; percent of population).

OVA sensitization resulted in AHR to MCh in the OVA exposed group compared to the Alum controls (Figure 2, top panel; *P* < 0.05). There was a tendency for changes in both the net WBC content of systemic blood and BAL consistent with pulmonary inflammation in OVA-sensitized mice; however, this did not reach statistical significance. Differential WBC analysis of the BAL fluid is consistent with a predominantly eosinophilic inflammation in OVA-sensitized mice compared with Alum controls (Figure 2, bottom right panel).

Systemic (right heart) injections of 50 mg/kg OVA in a separate group of sensitized mice caused a 62 ± 14% (mean ± SEM) increase in R_RS_ from baseline (1.3 ± 0.4 cm H_2_O/mL/s; mean ± SEM), which was consistent with the MCh response to 3–10 μg/kg in the same group (R_RS_ increases of 29 ± 7 and 89 ± 20%, respectively).

### Physiologic impact of OVA sensitization

**Figure 3:**
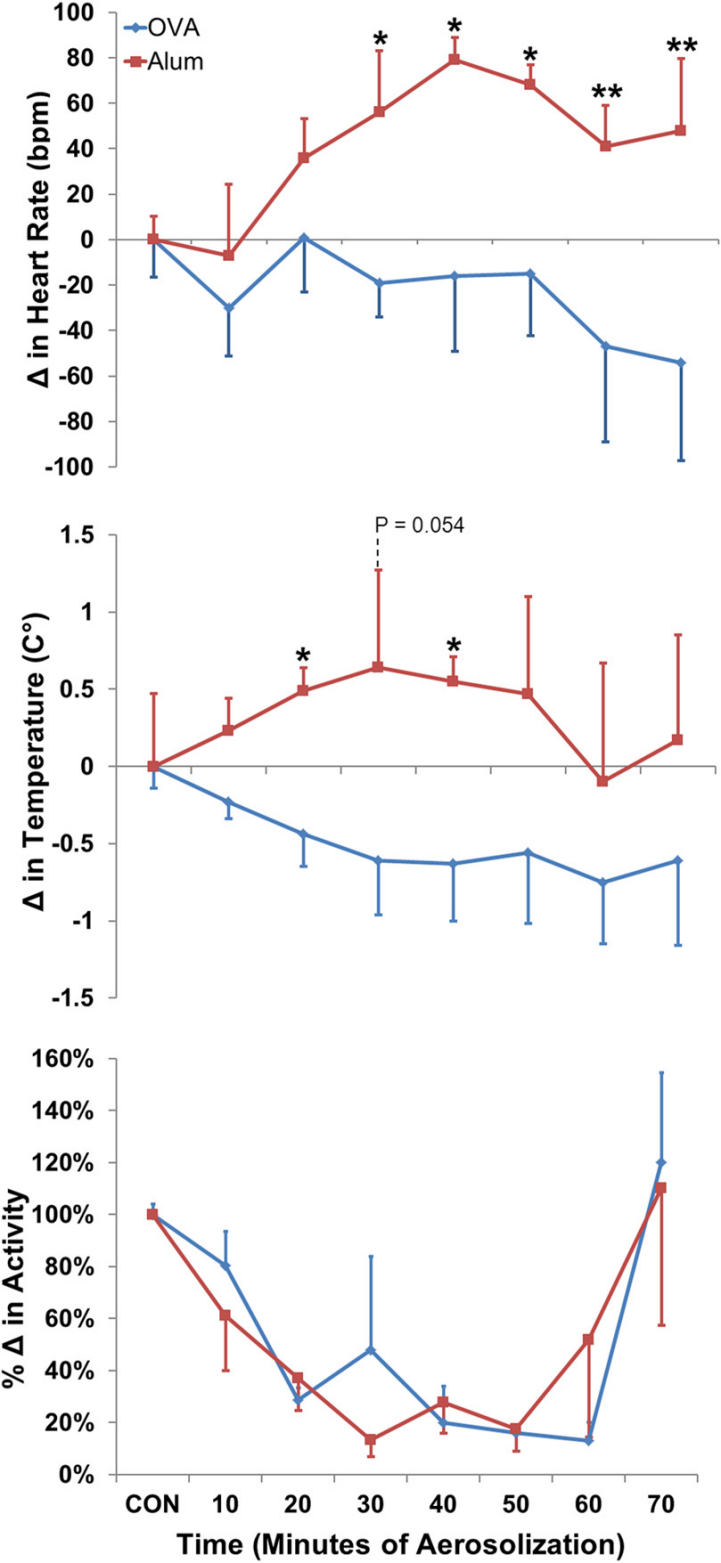
**Change in HR (top panel), T_B_ (middle panel), and activity (bottom panel) during aerosol challenge.** The baseline control values (CON) were calculated based on the means of HR, T_B_, and activity for the 40 min directly preceding Ag challenge. OVA-sensitized mice displayed a decreased HR and T_B_ profile compared with Alum control mice (both *P* ≤ 0.001 via Two-Way ANOVA, activity: NSD; Holm-Sidak post-hoc analyses: ^*^P < 0.05; ^**^P < 0.01).

Modest changes in HR, T_B_ and activity observed within each group during challenge were not statistically different from pre-aerosol baseline. However, analyses of HR, T_B_ and activity were different between groups and revealed that OVA sensitization attenuated the trend toward increased HR and T_B_ relative to baseline that was observed in the Alum group (Figure 3, *P* ≤ 0.001; maximum positive change in HR for Alum = 80 ± 10 bpm, for OVA = 1 ± 23 bpm, mean ± SEM). There was no difference in the activity profiles between the two groups during aerosol challenge (*P* > 0.05).

**Figure 4:**
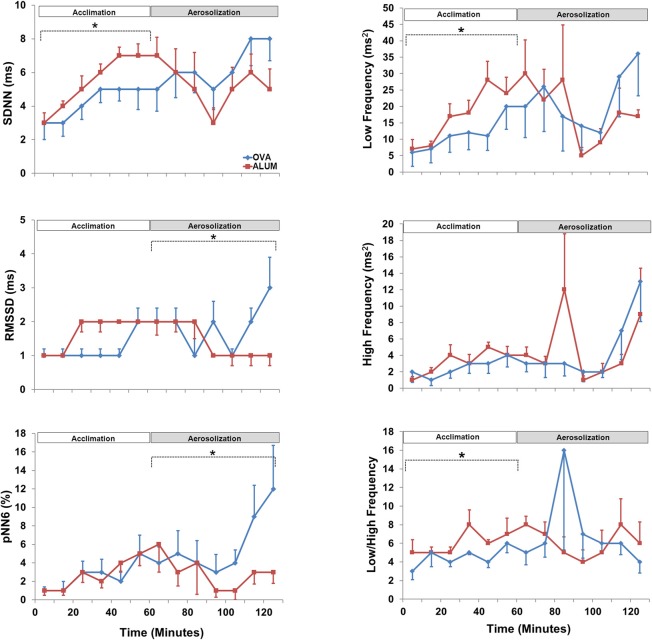
**Heart rate variability *prior* to and during aerosol challenge.** The acclimation control period (0–60 min) and aerosol challenge (70–130 min, to accommodate sampling) were independently analyzed by Two-Way ANOVA and Holm-Sidak post-hoc analyses; ^*^P < 0.05. During acclimation, OVA-sensitized mice displayed decreased SDNN (*P* < 0.05), LF (*P* < 0.025), and LF/HF (*P* < 0.05) compared with Alum control mice. During aerosolized Ag challenge, RMMSD (*P* < 0.05) and pNN6 (*P* < 0.05) were significantly increased in OVA-sensitized mice compared to the Alum controls.

HRV analysis in both time- and frequency-domains (SDNN, RMSSD, pNN6, LF, HF, and LF/HF) was analyzed separately for the acclimation and aerosol challenge (each 1 h). During the acclimation control period, three of the HRV variables were significantly decreased in the OVA-sensitized mice compared with Alum controls; specifically, SDNN (*P* < 0.05), LF (*P* < 0.025), and LF/HF (*P* < 0.05) (Figure 4). The remaining HRV variables were not significantly different, although they tended to be reduced in comparison with Alum control mice. During aerosol challenge, RMSSD (*P* < 0.05) and pNN6 (*P* < 0.05) were significantly elevated in OVA-sensitized mice (Figure 4) compared to Alum controls, whereas SDNN, LF, HF, and LF/HF did not vary significantly between groups (*P* > 0.05).

**Figure 5:**
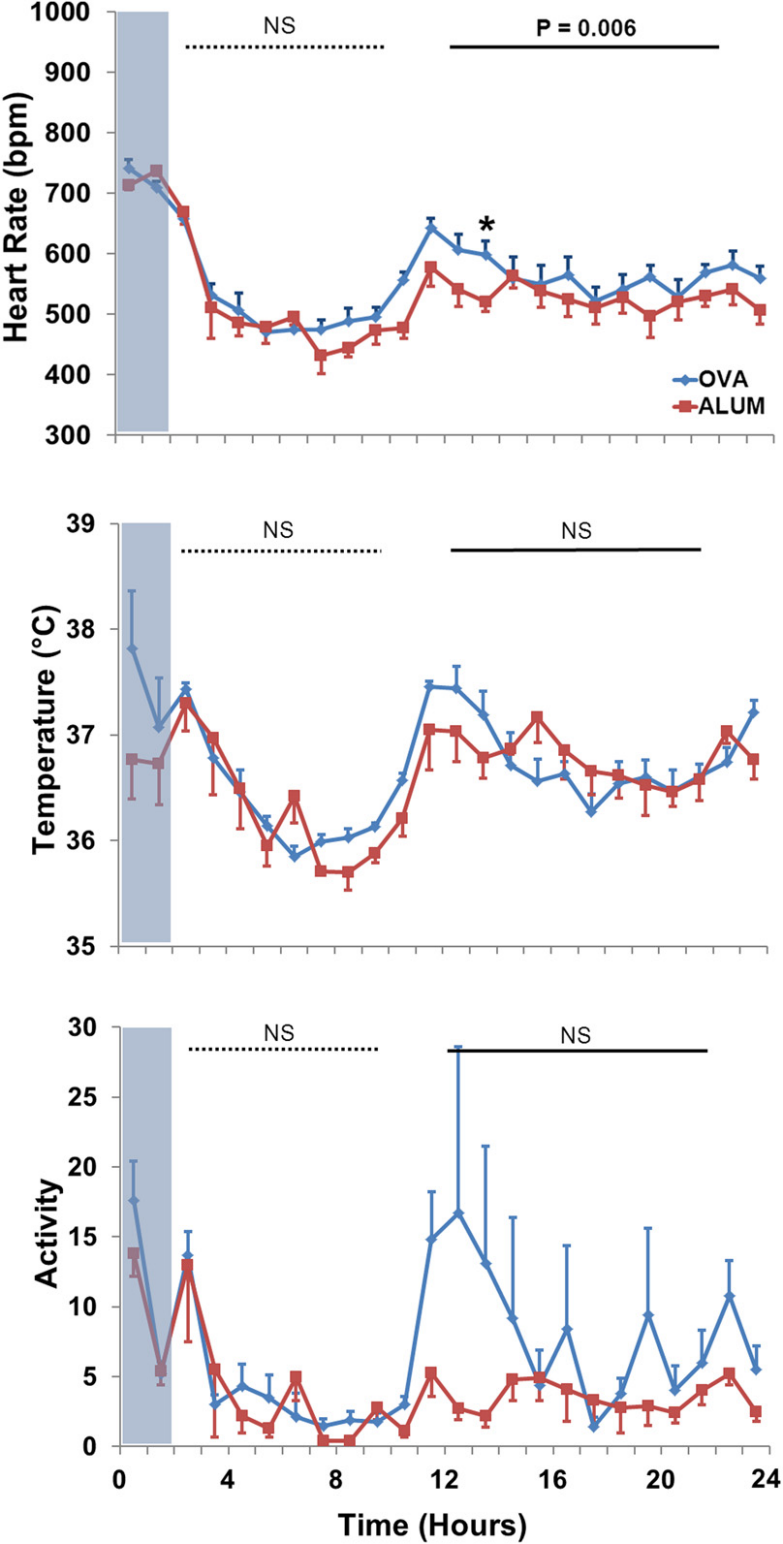
**HR (top panel), T_B_ (middle panel), and activity (bottom panel) during the light and dark cycles immediately following Ag challenge.** HR was increased in OVA-sensitized mice during the dark cycle (13–22 h) following Ag challenge (*P* < 0.01; Two-Way ANOVA with Holm-Sidak post-hoc analyses; ^*^P < 0.05).

Analysis of 3–24 h post-aerosol challenge revealed that HR and activity were elevated in OVA-sensitized mice compared with Alum mice (*P* ≤ 0.001 and *P* = 0.008, respectively; data not shown). Separate analysis of the light/dark cycle following sensitization (Figure 5) revealed that OVA sensitization was associated with an elevation in HR relative to Alum controls during the dark cycle (*P* < 0.01). There were no significant differences in T_B_ or activity between groups for either of the independently analyzed light or dark cycles (*P* > 0.05).

**Figure 6:**
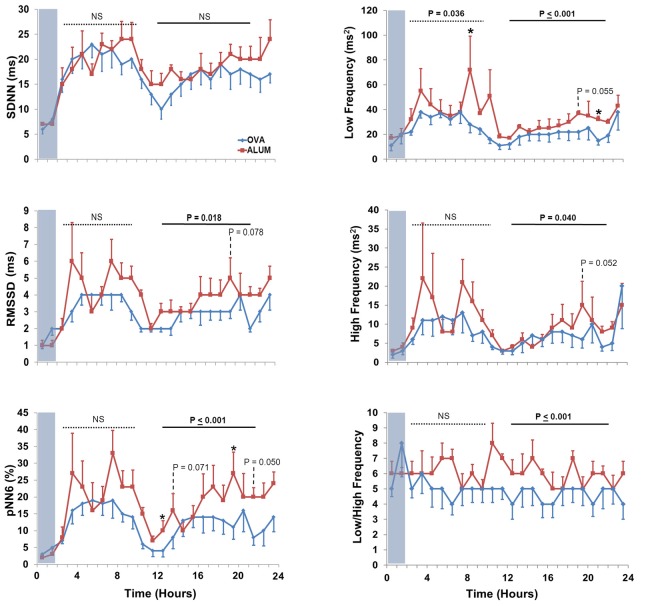
**The effect of OVA sensitization on HRV during the light and dark cycles immediately following aerosol challenge.** During the light cycle, OVA sensitization resulted in decreased LF relative to Alum controls (3–10 h; *P* < 0.05). During the dark cycle, OVA sensitization resulted in decreased RMSSD (*P* < 0.025), pNN6 (*P* ≤ 0.001), LF (*P* ≤ 0.001), HF (*P* < 0.05), and LF/HF (*P* ≤ 0.001; Two-Way ANOVA with Holm-Sidak *post-hoc* analyses; ^*^P < 0.05).

Six HRV parameters were analyzed for both the light and dark cycles (Figure 6). During the light cycle, LF was decreased in OVA-sensitized mice (*P* = 0.036), whereas five of the six HRV parameters were decreased during the dark cycle for OVA-sensitized mice (RMSSD, *P* = 0.018; pNN6, *P* ≤ 0.001; LF, *P* ≤ 0.001; HF, *P* = 0.040 and LF/HF, *P* ≤ 0.001).

## Discussion

The use of telemetered biological signals from chronically instrumented mice, coupled with the translation of HRV methodology to murine models (Thireau et al., 2008), provides a powerful experimental model to assess autonomic control *in vivo*. A primary outcome of our study was the demonstration of the viability of this approach, which incorporates abdominal implantation of the telemetry transmitter, to characterize the integrative biology/phenotype of the OVA allergen sensitization model. To our knowledge, the present study is the first to examine the murine integrative phenotype associated with the OVA model, as reflected by HR, T_B_ and HRV measurements, which are linked to autonomic nervous system (ANS) control (Thayer et al., 2011). We found that OVA sensitization was associated with altered control/baseline HRV *prior* to the aerosol challenge, as reflected by decreased SDNN, LF, and LF/HF. This suggests that early sensitization, *prior* to airway remodeling, may already exert subtle influences on autonomic control. Control of both HR and T_B_ during aerosol challenge, as well as HR in the circadian dark cycle subsequent to aerosol challenge, was altered in OVA-sensitized mice relative to the alum controls. OVA sensitization was associated with altered HRV (increased RMSSD and pNN6) during aerosolization challenge, as well as during the dark phase subsequent to aerosol challenge (decreased RMSSD, pNN6, LF, HF, and LF/HF compared to controls). Thus, our findings suggest that subtle changes in autonomic control are initiated early in the development of Ag sensitization associated with airway hyperresponsiveness (AHR).

### Methodological considerations

The use of a validated method to measure an enhanced R_RS_ response to MCh (Volgyesi et al., 2000; Ewart et al., 2001; Walker et al., in this issue) indicated the presence of AHR due to the OVA protocol [Figure 2, top panel; (Kumar et al., 2008)]. Since our model required inhalation of OVA aerosol, it is possible that the changes we observed in HRV and other variables during and/or after the third and final OVA exposure were a consequence of a bronchoconstrictor response to inhaled antigen, rather than overall changes in autonomic control associated with early allergic sensitization/AHR. Several observations suggest this may be unlikely in conscious mice. First, OVA-sensitized mice displayed changes in baseline HRV that were present *prior* to the final OVA exposure and the onset of presumed changes in bronchomotor tone. Based on the current literature, it is not clear if, when, or to what degree OVA inhalation causes bronchoconstriction in the murine model. For example, AHR to contractile agonists has been demonstrated to occur *prior* to the presence of a bronchoconstrictor response to inhaled OVA in sensitized mice (Neuhaus-Steinmetz et al., 2000). Additionally, bronchoconstriction to inhaled OVA has been shown to require multiple exposures (Zhang et al., 1997; Neuhaus-Steinmetz et al., 2000), with the magnitude of bronchoconstriction increasing with additional exposures (Neuhaus-Steinmetz et al., 2000). Secondly, putative methods to measure bronchoconstriction non-invasively in conscious animals, such as enhanced pause (Penh) or peak expiratory flow measurements, do not provide accurate or robust measurements of bronchoconstriction (see Walker et al., this volume, for review). As a result, data do not appear to be available to demonstrate that the third OVA exposure caused acute bronchoconstriction. This is an important unanswered question in the literature on murine models, which is further complicated by the wide array of variable murine OVA models documented in the literature (Holgate, 1999; Pabst, 2002; Kips et al., 2003; Epstein, 2004; Chang and Mitzner, 2007; Zosky and Sly, 2007; Kumar et al., 2008; Nials and Uddin, 2008; Zosky et al., 2008; Allen et al., 2009).

In order to gain some insight into bronchoconstrictor sensitivity to antigen, we performed additional studies on sensitized animals in which the R_RS_ response to vascular (right heart injection) delivery of OVA (50 mg/kg) was measured in anaesthetized, mechanically ventilated mice. Single high dose OVA increased Rrs comparable to that seen for low dose MCh (see section “Results”). This exposure is likely to be far greater than the dosage of OVA delivered through inhalation, where upper airway impaction, large airway branch-point deposition and the presence of a robust airway epithelial barrier (Hogg, 1982; Turi et al., 2011) all conspire to reduce the dosage of agonist delivered to submucosal airway smooth muscle sites. Thus, it is possible that the final inhaled OVA exposure may not have significantly altered R_RS_ or induced bronchoconstriction. If so, then changes in variables such as HR, HRV, T_B_ and activity, during and after exposure (i.e., subsequent 22 h) may be a reflection of the development of sensitivity in the OVA-model, and the underlying link between inflammation and the autonomic nervous system. Unfortunately, our data do not allow us to address the above questions definitively; however, they identify an important question for subsequent investigation.

### Early sensitization and physiologic outcomes

The functional change in AW responsiveness is accompanied, and arguably caused, by inflammatory changes, such as the localized infiltration of eosinophils into the AWs (Jacoby et al., 2001). We observed a trend (not statistically different) with respect to inflammatory mediators within the WBC content and populations of systemic blood and BAL fluid of OVA-sensitized mice, which is consistent with pulmonary inflammation, eosinophilia and the early nature of the sensitization. The statistical outcome may have also been influenced by group size.

Our acute model of OVA-induced AHR likely recreated the earliest functional stage of disease, *prior* to the remodeling routinely described in chronic asthma (Joos et al., 2000; Holgate, 2008; Allen et al., 2009). Although AW function was directly assessed 22 h subsequent to Ag challenge, as mentioned above we did not directly assess the presence or absence of early- and/or late-phase reactions (Boulet et al., 1999; Holgate, 2008). However, HRV analysis, in concert with analyses of HR, T_B_ and activity, suggests the impact of Ag exposure resulted in an altered overall autonomic profile for OVA-sensitized mice. Indeed, OVA-sensitized mice displayed a decreased HR and T_B_ profile during the Ag challenge compared to the Alum group (Figure 5). These differences were not activity related since the profiles were the same between groups. The changes in HR persisted after Ag challenge, with OVA-sensitized mice displaying increased HR during the dark cycle, over 10 h following the aerosolization exposure.

### HRV analyses

Quantifiable, integrative measures of end-organ responsiveness, changes in HR (gross autonomic balance) and HRV have been suggested to be robust indices of cardiac health in human subjects (Zwiener et al., 2002; Sandercock and Brodie, 2006; Chattipakorn et al., 2007; Thireau et al., 2008). We performed the two predominant types of HRV analyses: time-domain, which reflects the statistical analysis of IBIs in parameters such as SDNN, RMSSD, and pNN6, and frequency-domain, based on the spectral analysis of IBIs reflected by LF, HF, LF/HF (Figures 4, 6) (E.S.C and N.A.S.P.E. Task Force, 1996; Thireau et al., 2008). In humans, decreases in HRV are associated with worse prognoses, and HRV is an independent predictor of cardiac morbidity and mortality, and a useful tool for non-invasively measuring changes in sinoatrial (SA) ANS activity in health and disease (Tarkiainen et al., 2005; Parati et al., 2006; Sandercock and Brodie, 2006; Chattipakorn et al., 2007; Thireau et al., 2008). We adopted the guidelines developed by Thireau et al. for HRV analysis without correction for the potential impact of respiration on HRV (Thireau et al., 2008). More recently, it has been suggested that the presence of bi-directional cross-talk between the cardiovascular and respiratory systems, or cardiorespiratory coupling, makes correction for respiration less convincing in the examination of HRV (Tzeng et al., 2003; Thayer et al., 2011). Nevertheless, additional studies are required to assess the potential changes in pattern of breathing and lung volume, which may have occurred during OVA exposure.

HRV indices may represent specific components of the ANS; some investigators believe that time-domain HRV parameters can be ascribed to net autonomic activity (SDNN), short-term HRV (RMSSD), and PSNS activity (pNN6, or its human equivalent pNN50) (Thireau et al., 2008; Tascilar et al., 2009). Others have reported both RMSSD and pNN6 to primarily reflect short-term HR modulation, or PSNS activity, with SDNN reflecting the balance between the PSNS and SNS, or a combination of short-term (e.g., respiratory) and long-term (e.g., circadian) factors (Stein et al., 1994). Similarly, while HF HRV fluctuations are generally considered to be a reasonable approximation of PSNS activity, LF HRV fluctuations have been reported to reflect both SNS as well as a combination of SNS and PSNS activity (Pomeranz et al., 1985; Parati et al., 2006). Despite suggestions that HRV reflects integrated ANS activity at the level of the SA node, caution should be exercised in ascribing a link between specific components of the ANS and individual HRV parameters variables (Parati et al., 2006; Taylor and Studinger, 2006a,b). Interestingly, studies investigating HRV in humans have shown PSNS activity to be inversely related to systemic inflammation, as assessed by C-reactive protein levels (Thayer and Fischer, 2009). This association was stronger in females than in males. The present study used only male mice, and it remains to be seen if such associations are present between sexes for mice.

### Control and aerosol exposure

We investigated HRV *prior* to, during, and subsequent to aerosol challenge. During the hour *prior* to aerosol challenge (acclimation), three indices of HRV (SDNN, LF, LF/HF; all *P* < 0.05) were decreased in OVA-sensitized mice. These results suggest that there is a small, but significant alteration in ANS activity *prior* to Ag challenge, presumably due to the effects of sensitization alone. This decrease in activity may be linked to the sympathetic nervous system, as LF as well as LF/HF and SDNN were decreased in OVA mice, while RMSSD, pNN6, and HF were unchanged between groups.

During Ag challenge, RMSSD and pNN6 were increased (*P* < 0.05) in OVA mice, which is consistent with the time domain HRV results of Tascilar and colleagues for symptomatic pediatric patients with allergic rhinitis (Tascilar et al., 2009). We speculate the increase in RMSSD and pNN6 may be linked to an increase in pulmonary cholinergic tone, as supported by AHR during the MCh challenge. Reports of heightened cardiac vagal reactivity, despite insignificant alteration in basal vagal tone, have been reported in asthmatic patients (Lewis et al., 2006). Additionally, early studies reported increased indices of HRV in pediatric patients with asthma (Tokuyama et al., 1985). Increases in PSNS activity may reflect the increase in cholinergic bronchoconstrictive activity during an acute asthma exacerbation. Whether pathogenic changes in bronchial vagal tone can independently translate into changes in cardiac vagal tone is somewhat controversial, with evidence in support either enhanced or unaltered cardiac vagal activity in cases of high bronchial vagal activity (Lewis et al., 2006). Our murine model differed from Tascilar's human population in regards to its frequency domain responses; no differences in frequency domain parameters were observed in the present study, whereas increased HF and decreased LF/HF were reported in the above-mentioned study (Tascilar et al., 2009). It remains to be seen whether the parallelism observed in the time domain results is maintained in comparisons with antigen exposures for human asthma or AHR.

HR, T_B_, and activity were further investigated during the light and dark cycles following aerosol challenge, with OVA-sensitized mice displaying increased HR compared with Alum mice during the dark cycle 13–22 h after protocol initiation, over 10 h after aerosolization challenge. This further supports the concept of altered ANS control linked to sensitization, not exclusively during acute Ag exposure, as sensitized mice displayed persistent changes from Alum controls well after the aerosolization period. Analysis of HRV parameters in the light/dark cycles subsequent to the aerosol challenge period revealed that sensitized mice displayed decreased HRV in all, but one (SDNN) of the HRV indices during the dark cycle (during the light cycle only LF was decreased in OVA-sensitized mice). These results are consistent with studies demonstrating a correlation between absolute HR and asthma severity, where HR increases compared to controls as asthma severity increases (Kazuma et al., 1997). Further, our results are consistent with observations of decreased HRV during high absolute HR (Tascilar et al., 2009) There were no differences in activity, T_B_ or light-cycle HR. As HRV has been shown to be influenced by physical activity level (e.g., moderate-to-vigorous activity may help normalize HRV profiles in asthmatic subjects) the lack of difference in activity profiles of the two groups in the present study suggest the changes we observed in HRV are attributable to factors other than physical activity level (Tsai et al., 2011). The increased HR observed in sensitized animals, which is consistent with high resting HR in asthmatic patients, could be due to increased cardiac SNS tone, higher levels of circulating catecholamines, withdrawal of PSNS tone, or some combination of the aforementioned (Lewis et al., 2006). Further studies characterizing the nature and time-course of HRV changes in asthma disease progression or in animal models of asthma, are required to elucidate the physiologic and molecular mechanisms responsible.

In summary, to our knowledge this is the first study to document the use of chronic instrumentation and ANS assessment via HRV in allergically sensitized mice before, during and after aerosol challenge at early disease onset. Chronic instrumentation with radiotelemetry devices provides a robust way of gathering integrative physiological data to monitor the ANS phenotype of allergic sensitization. In the presence of mild sensitization, as evidenced by AHR, sensitized mice exhibit significant changes in HRV during acclimation, aerosol exposure and for the 22 h following aerosol challenge. Most strikingly, HRV is depressed in sensitized mice *prior* to Ag challenge, and exhibits a prolonged reduction in almost all indices for 10 h after aerosol exposure (i.e., during the dark/active cycle of the mice). This suggests that sensitization independently impacts on ANS activity, as measured through HRV analysis. Further studies are required to elucidate the integrative impact of sensitization, and the putative mechanisms responsible for these alterations.

### Conflict of interest statement

The authors declare that the research was conducted in the absence of any commercial or financial relationships that could be construed as a potential conflict of interest.

## Abbreviations

AHR: Airway Hyperresponsiveness
Alum: Alum Adjuvant Control Group
ANS: Autonomic Nervous System
AW: Airway
BAL: Bronchoalveolar Lavage
HF: High Frequency Power
HR: Heart Rate
HRV: Heart Rate Variability
LF: Low Frequency Power
MCh: Methacholine
OVA: Ovalbumin
P_AW_: Airway Pressure
pNN6: Percentage of consecutive normal R-R intervals differing by more than 6 ms
RMSSD: Square root of the mean of the sum of squares of successive differences between normal R-R intervals
R_RS_: Total Respiratory Resistance
SDNN: Standard deviation of normal R-R intervals
T_I_: Inspiratory Time
T_B_: Body Temperature
V_T_: Tidal Volume
WBC: White Blood Cell.
